# Transforming Medical Education to Make Patient Safety Part of the Genome of a Modern Health Care Worker

**DOI:** 10.2196/68046

**Published:** 2025-01-17

**Authors:** Peter Lachman, John Fitzsimons

**Affiliations:** 1 Quality Improvement Department, Royal College of Physicians of Ireland, 19 South Frederick Street, Dublin, D02 X266, Ireland, 353 0862334277; 2 Children’s Health Ireland at Temple Street, Dublin, Ireland

**Keywords:** patient safety, psychological safety, medical curriculum, professional competence, clinical competence

## Abstract

Medical education has not traditionally recognized patient safety as a core subject. To foster a culture of patient safety and enhance psychological safety, it is essential to address the barriers and facilitators that currently impact the development and delivery of medical education curricula. The aim of including patient safety and psychological safety competencies in education curricula is to insert these into the genome of the modern health care worker.

It has been over 25 years since the beginning of the active development of the patient safety movement, during which the theories and methods of patient safety science have evolved. We now understand the key drivers and practices for safer care [1], and while there have been many achievements and successes in implementation, transfer to different contexts, reliability, and sustainability remain challenging.

One of the underlying problems is that the health care workforce has limited training in the theories and methods of patient safety and insufficient training in improvement or implementation science. Developing sustainable changes in the way we approach patient safety requires radically rethinking how we educate the health care workforce of the future, so that patient safety becomes integrated into the way they work, that is, part of the genome of a modern health care worker.

In the past, patient safety was presumed to be synonymous with being a professional, so it was implicit that on completing medical or nursing education, one would be safe. This clearly is not the case. The paper by Carillo et al [2] approaches this challenge from the perspective of the critical practice of psychological safety in the workforce as the foundation for safer care. They first assessed the current status of training programs for medical students and trainees across Europe, with regard to the acquisition of knowledge, skills, and attitudes about patient safety. This was followed by the development of a suggested set of competencies for psychological safety that should be acquired during training programs. The curriculum is a valuable addition to our understanding of foundations for safer care. The focus on psychological safety as a key competency of patient safety training, rather than a mere focus on knowledge, is a novel approach to the curriculum. Additionally, it can form the basis of a better response to the second victim following an adverse event. Several themes arose from the paper that should be considered.

First, the focus on psychological safety changes the focus of education, shifting away from concentrating only on knowledge acquisition, as in other curricula such as the World Health Organization (WHO) [3] patient safety curriculum, which is under review. A modern patient safety curriculum needs to specify the learner outcomes of knowing what to do and how to generate feelings of being psychologically safe when applying safety science in the workplace to create safer clinical teams. This will be essential for the delivery of the WHO Patient Safety Global Action Plan [4].

Second, a patient safety curriculum cannot stand alone outside the wider concepts of quality in health care. This is an ongoing debate, but many other domains of quality impact the safe delivery of care. Therefore, a patient safety curriculum should be part of a comprehensive set of competencies that facilitate the implementation of patient safety improvement initiatives. Knowledge and skills of improvement methodology and implementation science are essential, and there are examples of frameworks that achieve this goal for comprehensive quality in health care [5]. Equally, psychological safety influences the success of improvement efforts, implementation efforts, and innovation, all of which depend on being able to speak openly and share new ideas without fear.

Third, it should be determined whether one can engender psychological safety via a training program alone and whether a program is the sole foundation on which a safe system can be built. Organizational culture is fundamental for psychological safety. Psychological safety can only thrive within a team or organization that has a culture of safety that includes a focus on communication, feedback, respect, and trust [6]. Although this is part of the program suggested, there is often a disconnect between the theoretical classroom and trainees’ lived experiences. Psychological safety requires positive team and organizational relationships that facilitate team members being safe [7]. The proposed framework for psychological safety includes structural, interpersonal, and individual factors that extend beyond education and depend heavily on leadership. Applying this in practice is challenging [8].

Fourth, to create a strategy for safer care delivery, we need to consider the reasons why medical education has not made patient safety an integral part of the curriculum, despite growing evidence of interventions that decrease harm and create a safer health care environment. Most academic institutions remain hierarchical and are steeped in the traditional medical model of teaching. Reasons for the reluctance to incorporate patient safety include lack of awareness of the emerging science, lack of leadership prioritization, curriculum overload, and competition with other emerging sciences [9]. For the proposed curriculum to succeed, these challenges need to be addressed head-on, and a radical rethink of medical education is required.

Finally, we need to consider the efficacy of patient safety training to make a difference. Two systematic reviews indicate the heterogeneity of papers that assess the effectiveness of patient safety education programs. The link between education and improved clinical outcomes is not strong [10]. There appears to be a disconnect between undergraduate patient safety training and what happens in the clinical setting [11]. This indicates the need for training programs to be integrated into postgraduate and undergraduate programs. It also suggests the need for early evaluation of any new program to ensure that what is imagined is being achieved.

In conclusion, Carillo et al [2] have shown a way forward for patient safety training. The challenge will be implementation within traditional medical education curricula. Perhaps the solution to this could be coproduced by educators, trainees, and patients rather than created by experts and mentors alone. Even though the goal must be transformation in how patient safety is considered within medical education, we can start to create the conditions for these competencies to thrive at our next classroom meeting, simulation session, team huddle, or handover. Imagine the power of a senior clinician openly sharing their vulnerability of not being able to know everything that is required to be safe, inviting respectful dissent, and graciously embracing difficult news. That is the improvement way!

## Abbreviations

WHO: World Health Organization
